# Protective Effects of *Codium fragile* Extract against Acetaminophen-Induced Liver Injury

**DOI:** 10.4014/jmb.2409.09061

**Published:** 2024-10-25

**Authors:** Yea-Lim Lee, Ji-Yun Lee, Joo-Woong Park, Jin Lee, Hyun-Hoo Lee, Dae-Hee Lee

**Affiliations:** 1 Nbio, Inc., Gangneung 25457, Republic of Korea; 2 Biostream Co., Ltd., Suwon 10442, Republic of Korea; 3 Department of Marine Bio Food Science, Gangneung-Wonju National University, Gangneung 25457, Republic of Korea

**Keywords:** Acetaminophen, *Codium fragile* extract, anti-inflammation, loliolide, liver injury

## Abstract

Acetaminophen (APAP) is a well-known analgesic used globally. Generally, APAP has been proven to be safe and effective at therapeutic doses; however, it can cause serious liver damage when administered at high levels. We prepared *Codium fragile* extract (CFE) using the seaweed *C. fragile* and confirmed that the CFE contains a substance called Loliolide with antioxidant activity. We performed the present study to determine whether CFE protects HEPG2 cells and BALB/c mice from oxidative stress-induced liver damage. We confirmed that CFE and Loliolide were non-cytotoxic and protected against liver damage by reducing the activities of ALT and AST, which were increased by APAP treatment, and that CFE reduced the mRNA expression of inflammatory cytokines TNF-α and IL-6 and inhibited the phosphorylation of ERK and p38 in HEPG2 cells as determined by RT-PCR and Western blot analyses. Furthermore, the TNF-α and IL-6 levels, which were increased after APAP treatment in BALB/c mice, decreased after CFE treatment. Therefore, we demonstrated that CFE exerts a protective effect against APAP-induced liver injury by suppressing the inflammatory response through anti-inflammatory activity. Our findings provide new perspectives for developing functional foods that utilize seaweeds to improve liver function.

## Introduction

The liver is one of the most important organs in the body because it regulates many physiological processes. The liver is responsible for various physiological functions such as metabolism, immune function, blood coagulation control, removal of toxic substances from the blood, and secretion and excretion [1]. The liver appropriately converts ingested food into the form of nutrients required by various tissues and transports the remaining waste products for processing. Particularly, the liver protects the body by destroying harmful substances that invade the body and by detoxifying toxins. Although liver cells have sufficient reserve functions to prepare for liver damage, it is difficult to recognize when they are damaged in large quantities and are impaired; therefore, it is very important to maintain normal liver function.

Acetaminophen (APAP) is one of the most commonly used painkillers globally. Generally, APAP was proven to be safe and effective at therapeutic doses through earlier research, although an overdose can cause serious liver damage and, in severe cases, death [2]. Since APAP currently exists in complexes with many drugs, excessive drug intake by individuals can lead to an APAP overdose. The main mechanisms that cause liver damage due to APAP overdose include inflammation, necrosis, and apoptosis [3].

Numerous studies found several cytokines upregulated during excessive APAP-induced toxicity and may contribute to APAP pathogenesis [4]. Additionally, TNF-α has been reported to play an important role in hepatocyte regeneration in several liver injury models [5]. Therefore, regulating the induced inflammatory cytokines is important for liver protection.

Seaweeds are rich sources of bioactive compounds with significant pharmacological potential [6]. Research on improving liver function using seaweeds with antioxidant activity is being conducted [7]. *Codium fragile*, a type of seaweed, is widely distributed in oceans globally, except in the polar regions, and is mainly found in temperate and subtropical regions [8]. It has great potential as a source of abundant bioactive compounds [9] and has been used as a food source and medicinal herb in Asian countries since ancient times.

Therefore, in this study, we aimed to confirm the protective effect of prophylactic extract against APAP-induced liver damage. Our findings may provide a new perspective for developing functional foods that utilize seaweeds to improve liver function. As such, developing healthy, functional food materials using seaweed may represent a strategy for improving people's health in countries with abundant seaweed resources. It has the potential to bring about a new approach in research materials, helping to enhance anticancer properties, boost immunity, and improve liver function.

## Materials and Methods

### Preparation of Codium Fragile Extract (CFE)

The *Codium fragile* was obtained from Goheung-gun, Jeollanam-do, Korea. The ethanol extract (CFE) of *Codium fragile* was prepared and supplied by Biostream Inc., (Republic of Korea). Briefly, the *C. fragile* were collected and washed with excess tap water and hot air-dried. The dried and powdered *C. fragile* was diluted 15 times with 50% ethanol and extracted at 60°C for 15 h. The extracted mixture was filtered using a filter press and concentrated by 15 brix, then sterilized at 80°C for 30 min. The extract was prepared in powder form using a lyophilizer (Ilshin Bio Base, Republic of Korea), tentatively named CFE (50% ethanol extract of *C. fragile*), and stored at 4°C until use. HPLC performed a chemical profile analysis of CFE.

### Identification of Analysis of Loliolide Content

Authentic Loliolide was purchased from Sigma (USA) and dissolved in methanol for a standard solution (1.0 mg/ml). Solutions were prepared at 40, 30, 20, 10, and 5 μg/ml concentrations to construct the calibration curve. To analyze Loliolide in *C. fragile* extract, the freeze-dried extract was diluted 10 times with deionized water and dissolved at 37°C for 1 h with sonication. One volume of methanol was added and the mixture was dissolved for 10 min with sonication. The resulting solutions were filtered with a 0.22 μm nylon filter and used for the HPLC analysis. The presence of Loliolide in *C. fragile* extract was analyzed using the HPLC system (1260 series, Agilent, USA). Samples were dissolved in methanol and fractionated on an XDB-C18 column (150 × 4.6 mm, 5 μm column, Agilent), and eluted at 0.7 ml/min in gradient mode with a mobile phase consisting of water (0.01%formic acid) and acetonitrile (100:0 to 0:100 for 24 min).

### Cell Culture and Treatment

The cells used in this study were HEPG2, a human hepatocellular carcinoma cell line, purchased from American Type Culture Collection (ATCC, HB-8065). 10% Fetal Bovine Serum (12483-020, Gibco, USA) and 1%Anti-Anti (15240-062 Gibco) were added to DMEM (Gendepot, CM002-050) medium and cultured in a CO_2_ incubator at 37°C. The cell density in all experiments was set at 80–90%, and liver damage was induced with APAP (A7085, Sigma Aldrich) after sample pretreatment. All the samples were processed after the culture medium was refreshed. APAP, Loliolide (SMB01001, Sigma Aldrich), and silymarin (Sigma Aldrich, S0292) were used after being dissolved in dimethyl sulfoxide (DMSO), and CFE was used after being dissolved in PBS. In all experiments, pretreatment using Loliolide (5 μg/ml and 10 μg/ml), Silymarin (20 μg/ml), and *CFE* (5 μg/ml, 10 μg/ml, 25 μg/ml, and 50 μg/ml) was performed. Then, APAP (20 mM) treatment was performed to induce hepatotoxicity.

### Cytotoxicity Assay

HEPG2 cells were seeded at 2 × 10^4^ cells per well in a 96-well plate and cultured for 24 h. After treatment with CFE (5, 10, 25, 50 μg/ml), Loliolide (5, 10, 20 μg/ml), and APAP (1, 5, 10, 20 mM) for 24 h, cell proliferation was measured by WST-1 analysis using Ez-cytox (EZ-500, Dogen Bio, Republic of Korea). After treatment with 10%Ez-cytox, cells were reacted for 2 h in a 5% CO_2_ incubator at 37°C. Optical density (OD) values were analyzed by measuring absorbance at 450 nm using a microplate reader.

### Cell Viability Assay (Inhibition of APAP-Induced Hepatotoxicity)

HEPG2 cells were seeded into 96-well plates at 2 × 10^4^ cells/well and cultured for 24 h. After 24 h of incubation, the cells were pretreated with CFE, Loliolide, and silymarin. After pre-treatment and incubation for 24 h, hepatotoxicity was induced by treatment with APAP for 24 h. The WST assay was performed using Ez-Cytox to measure cell proliferation. After 10% Ez-Cytox treatment, the reaction was for 2 h at 37°C in a 5% CO_2_ incubator. Optical density (OD) values were analyzed by measuring the absorbance at 490–500 nm using a microplate reader.

### Western Blot Analysis

HEPG2 cells were seeded in 60 mm2 cell culture dishes at 8 × 105 cells and cultured for 24 h. After 24 h of culture, the cells were pretreated with CFE, Loliolide, and Sliymarin before inducing liver damage with APAP. After harvesting the cells using trypsin-EDTA, the cells were centrifuged at 1,500 rpm for 5 min, the supernatant was removed, and the cells were washed with ice-cold PBS at 1,500 rpm for 5 min. After removing the supernatant, the cells were lysed using 2x sample buffer (100 μl). After heating for 5–7 min using a heating block, the cells were quantified using Pierce BCA Protein Assay Kits (23225, Thermal Fisher Scientific, USA). Equal amounts of protein samples were separated by SDS-PAGE and transferred to PVDF membranes using an electro-blotting apparatus (Bio-Rad, USA). Membranes were blocked in blocking buffer (TBS-T + 5% skim milk) for 1 h and incubated overnight at 4°C with primary antibodies against p-ERK (CST, 9101S), p-p38 (CST, 9211S), and GAPDH (CST, 5174S). Membranes were washed with TBS-T and incubated with HRP-conjugated secondary antibodies (CST, 7074S) for 1 h at room temperature. Protein expression was visualized using Pierce ECL Western Blotting Substrate (32109, Thermo Fisher Scientific) and an Amersham imager 680.

### RNA Isolation and Gene Expression Analysis

HEPG2 cells were seeded in 6-well plates at 4 × 10^5^ cells/well and cultured for 24 h. After 24 h of incubation, pretreatment with CFE, Loliolide, and Silymarin was performed before hepatotoxicity was induced with APAP. Then, the cells were harvested and RNA was isolated using TRIzol reagent (Invitrogen, 15596026, USA). The RNA concentration was measured using a microvolume spectrophotometer (KLAB, OPTIZEN NanoQ Plus), and cDNA was synthesized using a High-Capacity cDNA Reverse Transcription Kit (Applied Biosystems, 4368814, USA). To measure the expression of genes, the Applied Biosystems StepOnePlus Real-Time PCR System was performed using Power SYBR Green PCR Master Mix (Applied Biosystems, 4367659). The base sequences of the PCR primers used for these genes are shown in Table 1.

### Measurement of Aspartate Aminotransferase (AST) and Alanine Aminotransferase (ALT) Activities

The experiment was conducted following the instructions of the Aspartate Aminotransferase (AST/GOT) Activity Assay Kit (E-BC-K236-M, Elabscience, USA) or Alanine Aminotransferase (ALT/GPT) activity assay kit (E-BC-K235-M, Elabscience) protocol. A standard curve was drawn using the standard substances provided in the kit, and the ALT and AST activities in the serum were determined from the standard AST and ALT calibration curves.

### Design of In Vivo Experiments

Four-week-old male BALB/c mice were conducted in a sterile rearing facility after a 1-week adaptation period. Eight mice per group were divided into different groups, as shown in Table 2.

The mice were administered CFE orally at a low (100 mg/kg/day) and a high (200 mg/kg/day) for one week; the mice in the positive control group were administered 200 mg/kg/day of silymarin orally for one week. The mice in the non-treated group were administered 0.9% saline orally for one week. After fasting for 6 h, APAP was dissolved in 0.9% saline and administered orally at 300 mg/kg/day; serum samples were obtained via orbital blood collection 18 h after APAP administration. All animal experiments were performed according to the guidelines for the operation of the Experimental Animal Steering Committee (IACUC).

### Measurement of ALT and AST Activity in Mouse Serum

Serum samples were obtained by centrifuging mouse blood from the orbital vein at 1300 ×*g* for 20 min; these samples were used to measure the activities of ALT and AST, which are amino acid transferases. The ALT and AST activities were measured using a kit (Elabscience Bioscience Inc., USA). A standard curve was drawn using the standard substances provided in the kit; the ALT and AST concentrations in the serum were determined from the standard AST and ALT calibration curves.

### Measurement of Interleukin 6 (IL-6) Levels in Mouse Serum

The level of IL-6, a cytokine identified as a biomarker of the inflammatory response in liver damage, was measured in the serum obtained from mouse blood. The IL-6 levels were measured using the Quantikine ELISA Mouse IL-6 Kit (R&D Systems, USA). The procedure was performed according to the manufacturer’s protocol presented in the kit, and a standard curve was drawn using the standard materials provided. The concentration of IL-6 in serum was determined using a standard IL-6 calibration curve.

### Statistical Analysis

All data are represented by mean ± standard deviation (SD) in at least two independent experiments. GraphPad Prism 5 software was used for statistical analyses. For comparisons between groups, one-way ANOVA followed by Tukey’s post-hoc test was used. Additionally, a *t*-test was used to evaluate the significance of differences between the groups. The significance of the *p*-value values was evaluated as *p < 0.05, **p < 0.01, and ***p < 0.001.

## Results and Discussion

### Chemical Profile of *C. fragile* Extract Determined by HPLC Analysis

**Fig. 1:**
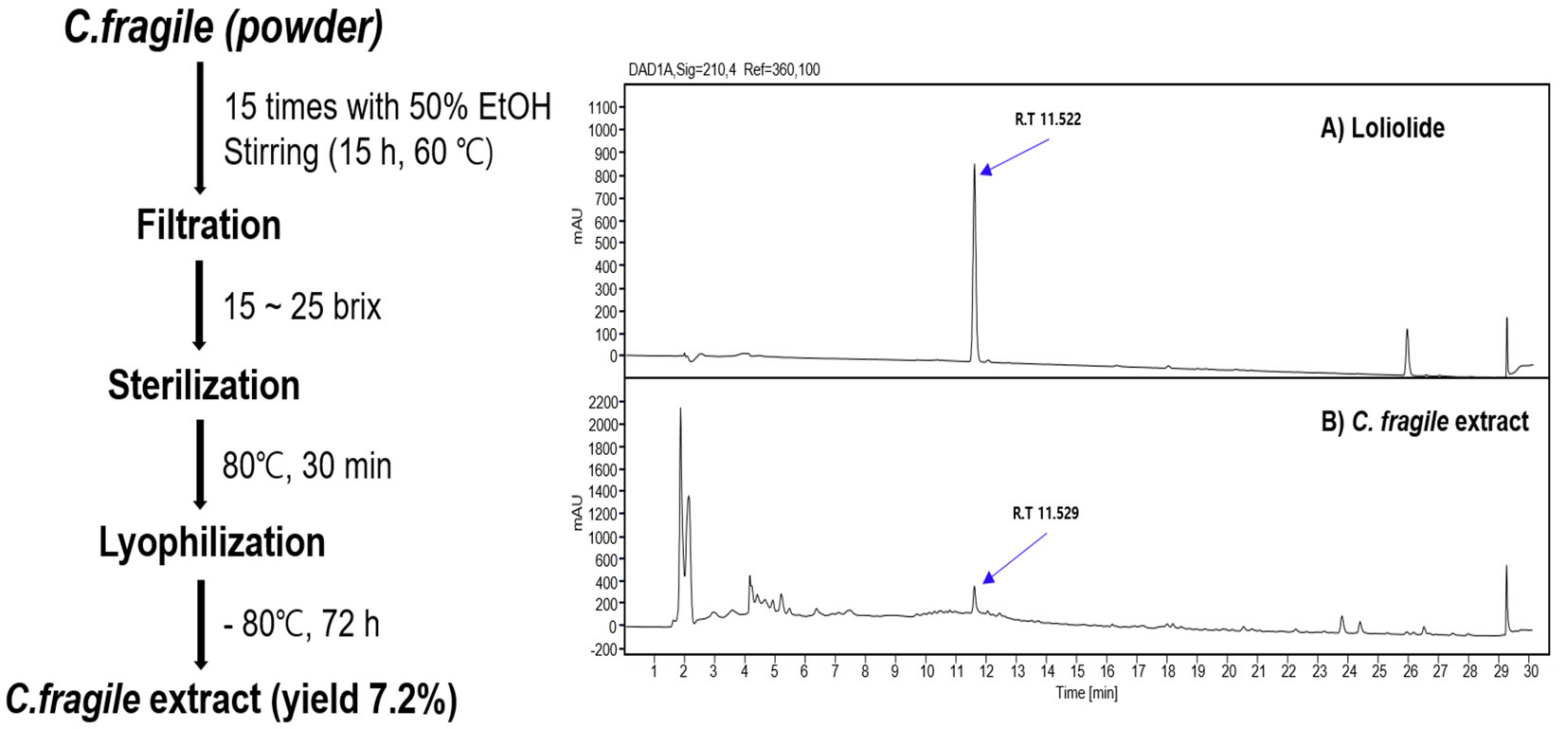
(A) Schematic of *Codium fragile* extract (CFE) production process. (B) HPLC analysis for Loliolide in CFE. CFE samples dissolved in 50% methanol were separated on an XDB-C18 column, detected by a UV detector at 210 nm, and quantified by comparing the peak area on the chromatogram set to the peak area of the Loliolide standard at 100% as described in the manuscript. *C. fragile*: *Codium fragile*, *C. fragile* extract: CFE.

Some natural chemicals such as siphonaxanthin, clerosterol oleamide, and Loliolide were detected in the CFE [10-12]. Loliolide was identified in 1964 by Hodges and Porte [13]. Loilolide was present in marine macroalgae and has been reported to have several biological activities [14, 15]. We analyzed Loliolide content in CFE as a marker compound for quality control. As shown in Fig. 1, HPLC analysis confirmed the presence of Loliolide in CFE, a 50% ethanol extract of the *C. fragile* containing 214.3 μg per g of CFE. (Fig. 1A and 1B)

### Cell Viability of CFE and Loliolide in HEPG2 Cells

**Fig. 2:**
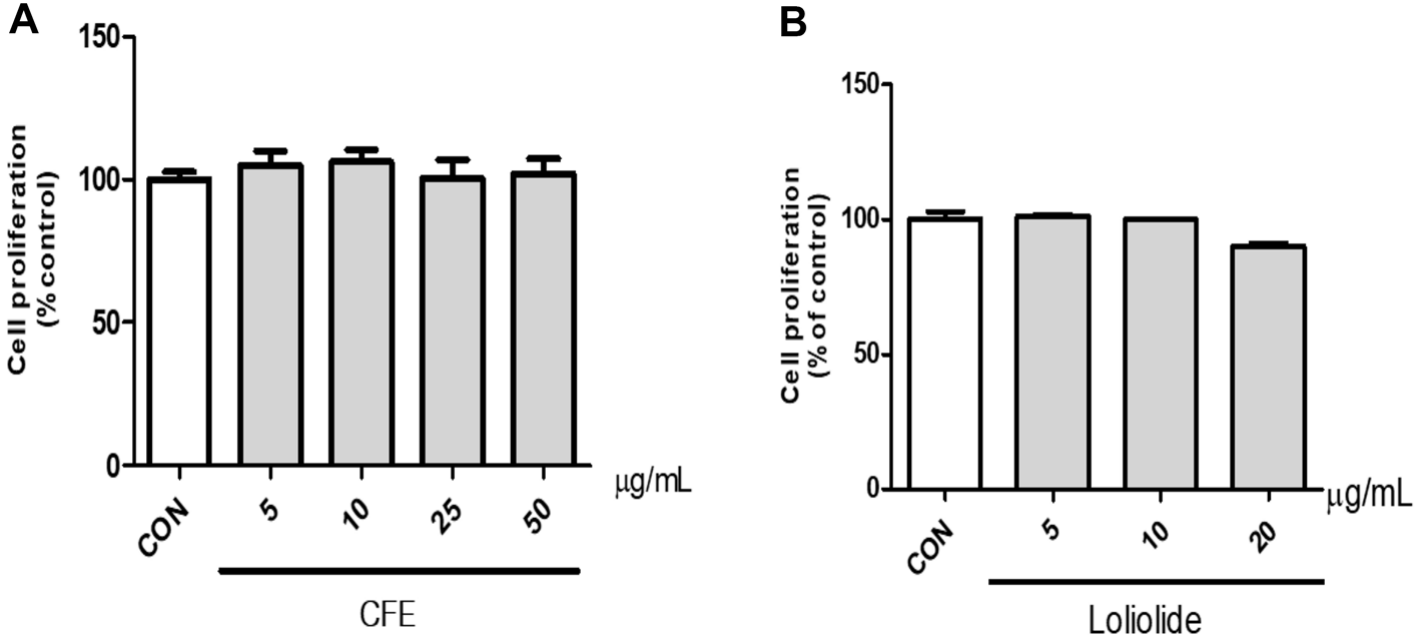
Cytotoxicity evaluation of CFE and Lolilolide in HEPG2 cells. (**A**) Cytotoxicity evaluation of CFE. (**B**) Cytotoxicity evaluation of Loliolide. Cells were treated with CFE (5–50 μg/ml) and Loliolide (5–10 μg/ml) for 24 h, respectively, and detected by the WST-1 method. Each CON was obtained in the absence of CFE and Lolilolide. Values are expressed as mean ± SME (*n* = 3).

Cell viability tests were performed to examine the cytotoxicity of CFE and Loliolide on HEPG2 cells. CFE did not show any toxicity at any concentration compared to the control (Fig. 2A). Loliolide showed about 12%decrease in viability compared to the control at a concentration of 20 μg/ml (Fig. 2B). Consequently, CFE was selected to examine the protective effect on APAP-induced liver cells in the concentration range of 5–50 μg/ml, and Loliolide was selected to examine the protective effect on APAP-induced liver cells in the concentration range of 5–10 μg/ml.

### CFE and Loliloide Exhibit Protective Effects in HEPG2 Cells Induced by APAP Damage

**Fig. 3:**
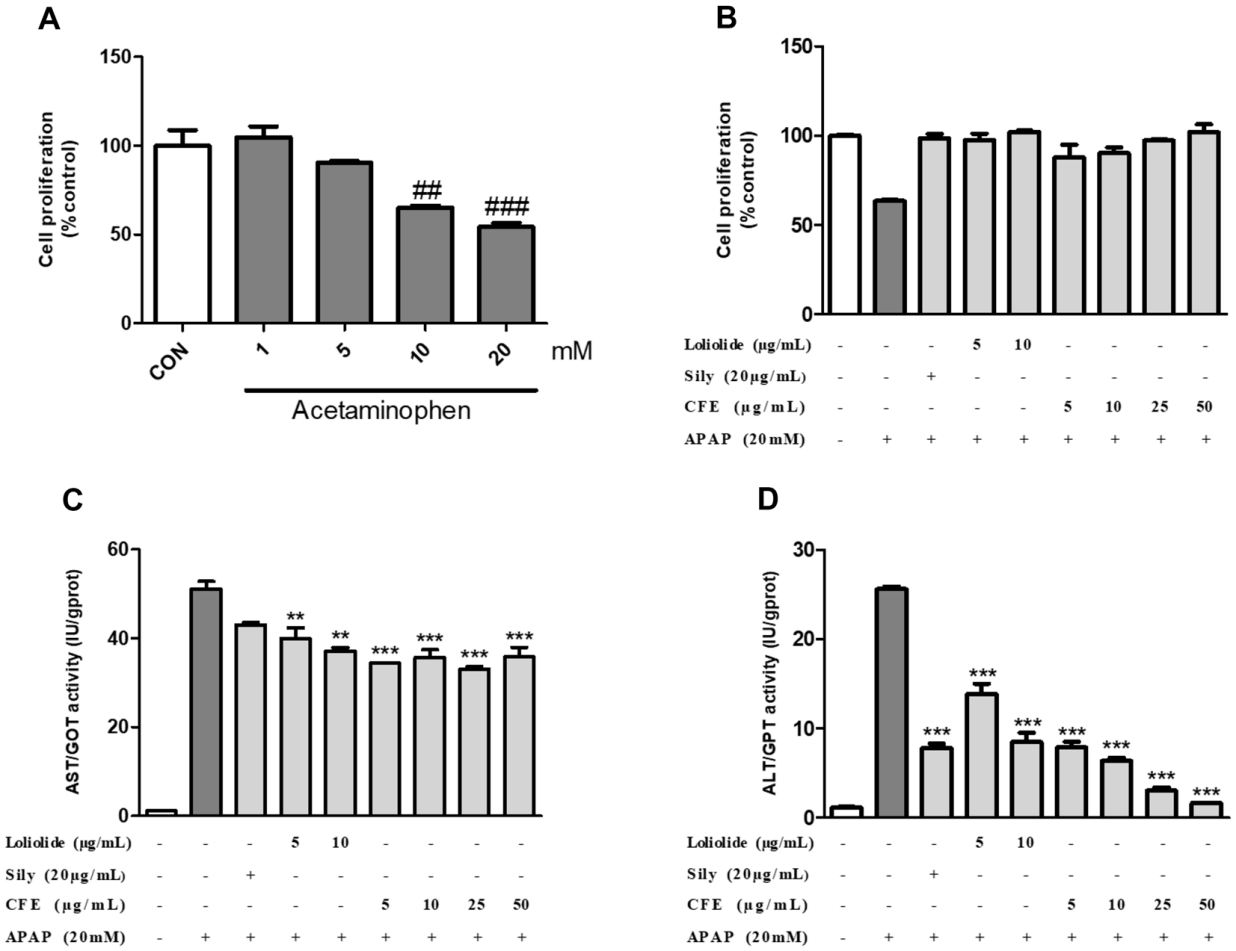
Protective effects of CFE and Loliolide on APAP-induced liver damage in HEPG2 cells. (**A**) Cytotoxicity evaluation of APAP. (**B**) Hepatoprotective effects of CFE and Lolilode as assessed by cell viability evaluation. (**C**) Hepatoprotective effects of CFE and Lolilode as assessed by AST activity. (**D**) Hepatoprotective effects of CFE and Lolilode as assessed by ALT activity. Cells were pretreated with CFE (5–50 μg/ml) and Lolilolide (5–10 μg/ml) for 24 h, and then the damage was induced by APAP and detected by the WST-1 method. Each CON was obtained in the absence of CFE, Lolilolide, and APAP. Cell viability values were expressed as means ± SME (*n* = 3), and AST and ALT activities were expressed as means ± SME (*n* = 2). ^##^*p* < 0.1 vs CON, ^###^*p* < 0.01 vs CON, *p < 0.5 vs APAP, **p < 0.1 vs APAP, ***p < 0.01 vs APAP. Sily: Silymarin.

HEPG2 cells are a classic hepatocyte model for studying pharmacological toxicity [16]. When HEPG2 cells were treated with APAP at concentrations ranging from 1 to 20 mM, we confirmed that significant damage occurred at concentrations of 10 and 20 mM (Fig. 3A). Consequently, we chose to induce damage at a concentration of 20 mM. After pretreatment with CFE and Lolilolide for 24 h and treatment with APAP for 24 h to induce liver damage, cell viability was measured. APAP showed a cell viability of approximately 60%, and CFE and Lolilolide showed cell viability equivalent to or superior to that of the positive control, Silymarin (Fig. 3B). Through the confirmation of cell viability, it was confirmed that CFE and Loliolide showed a hepatoprotective effect. The hepatoprotective effect of CFE and Loliolide was also confirmed through ALT and AST activities. ALT is an enzyme that transfers the amino group of alanine to a-ketoglutaric acid to produce glutaric acid, and AST is an enzyme that transfers the amino group of aspartic acid to a-ketoglutaric acid to produce glutamic acid [17]. AST and ALT are enzymes widely distributed in liver tissue and are known to be released when liver tissue damage is induced [18]. Therefore, ALT and AST are enzymes widely used as indicators of liver damage. In the case of AST, when APAP was treated alone, AST activity of about 50 IU/gprot was measured. Loliolide decreased AST activity to 39 IU/gprot at a 10 μg/ml concentration. CFE also confirmed that the average AST activity decreased to 39 IU/gprot in all concentration ranges (5–50 μg/ml) (Fig. 3C). When APAP was treated alone, ALT activity of about 25 IU/gprot was measured. Loliolide decreased ALT activity to 8 IU/gprot at a 10 μg/ml concentration. CFE also confirmed that ALT activity decreased to less than 2 IU/gprot from a concentration of 25 μg/ml or higher (Fig. 3D). These results suggest that CFE and Loliolide have protective effects in APAP-induced HEPG2 cells.

### CFE and Loliolide Inhibit the Expression of Inflammatory Cytokines and MAPK Activation in APAP-Induced Liver Injury HEPG2 Cells

**Fig. 4:**
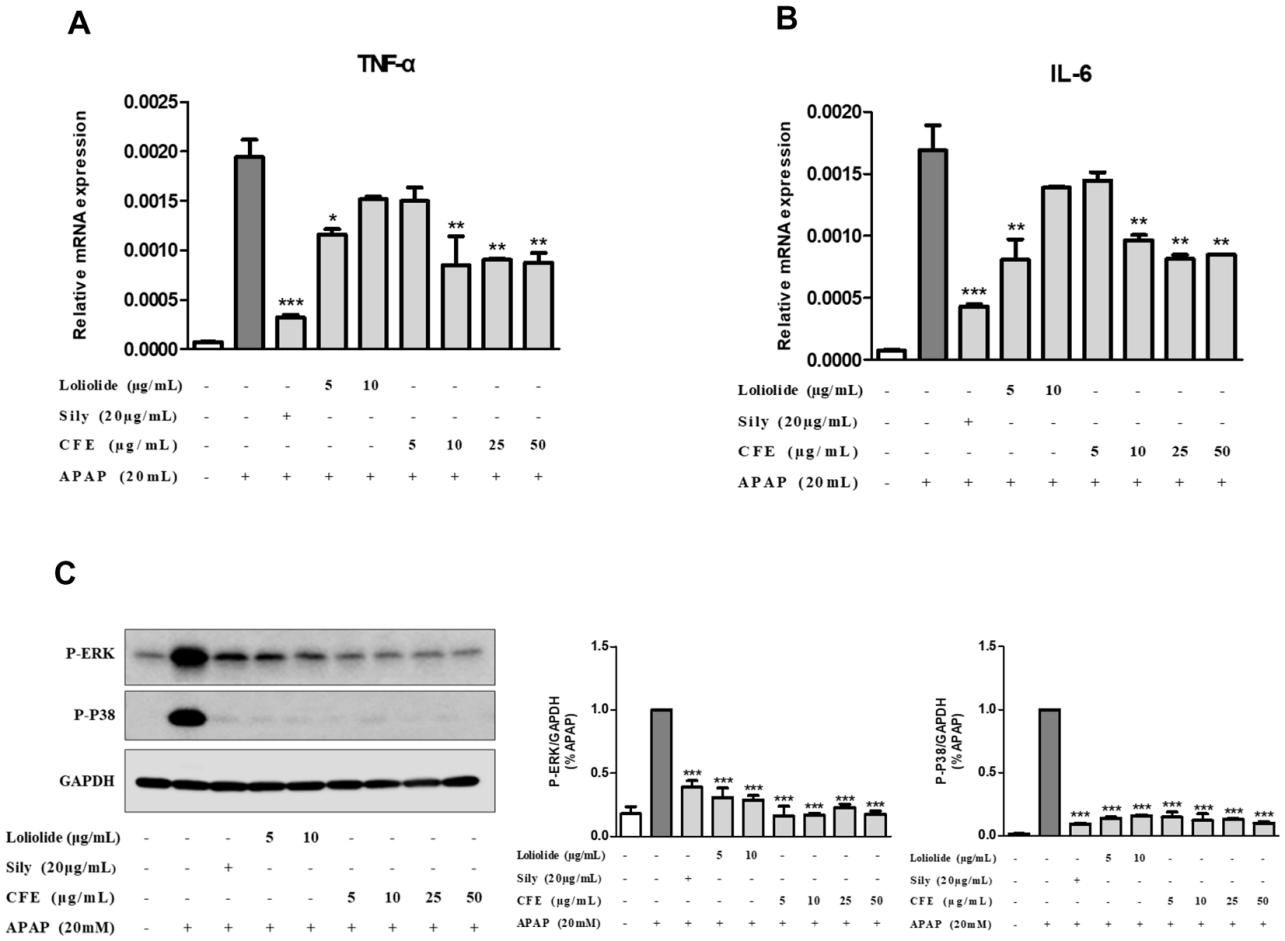
Anti-inflammatory effects of CFE and Loliolide in APAP-induced liver injury in HEPG2 cells. (**A**) mRNA expression of TNF-α, (**B**) IL-6 was measured by real-time PCR. (**C**) Phosphorylated proteins and relative protein levels were determined by Western blotting. Cells were pretreated with CFE (5–50 μg/ml) and Loliolide (5–10 μg/ml) for 24 h, and then APAP-induced injury was induced. Each CON was obtained in the absence of CFE, Lolilolide, and APAP. Values are expressed as mean ± SME (*n* = 2). *p < 0.5 vs APAP, **p < 0.1 vs APAP, ***p < 0.01 vs APAP. Sily: Silymarin.

An inflammatory response usually accompanies APAP-induced liver injury [19]. The inflammatory cytokine TNF-α is known as one of the earliest inducers of liver damage [20], and IL-6 is also one of the inflammatory cytokines immediately activated upon tissue damage [21]. As shown in Fig. 4A and 4B, APAP significantly increased the mRNA expression levels of TNF-α and IL-6. Compared with APAP, CFE and Loliolide showed decreased mRNA expression levels of TNF-α and IL-6. The MAPK pathway involves various cellular processes, including cell proliferation, apoptosis, and inflammatory responses. The activity of inflammatory cytokines is also regulated by the MAPK pathway [22]. Among the numerous signal regulatory molecules of the MAPK pathway, ERK and p38 are representative signal regulatory molecules. Signal transduction of phosphorylated ERK and p-38 is involved in the inflammatory response in MAPK [23]. In Fig. 4C, we confirmed that CFE and Loliolide significantly reduced APAP-induced phosphorylation of kinases, while APAP promoted phosphorylation of ERK and p-38. Therefore, these results showed that CFE and Loliolide exhibited anti-inflammatory effects by inhibiting the phosphorylation of inflammatory regulatory genes and inflammatory signaling regulatory molecules activated by APAP-induced liver damage.

### Confirmation of the Protective Effects of CFE against APAP-Induced Liver Damage in Mice

**Fig. 5:**
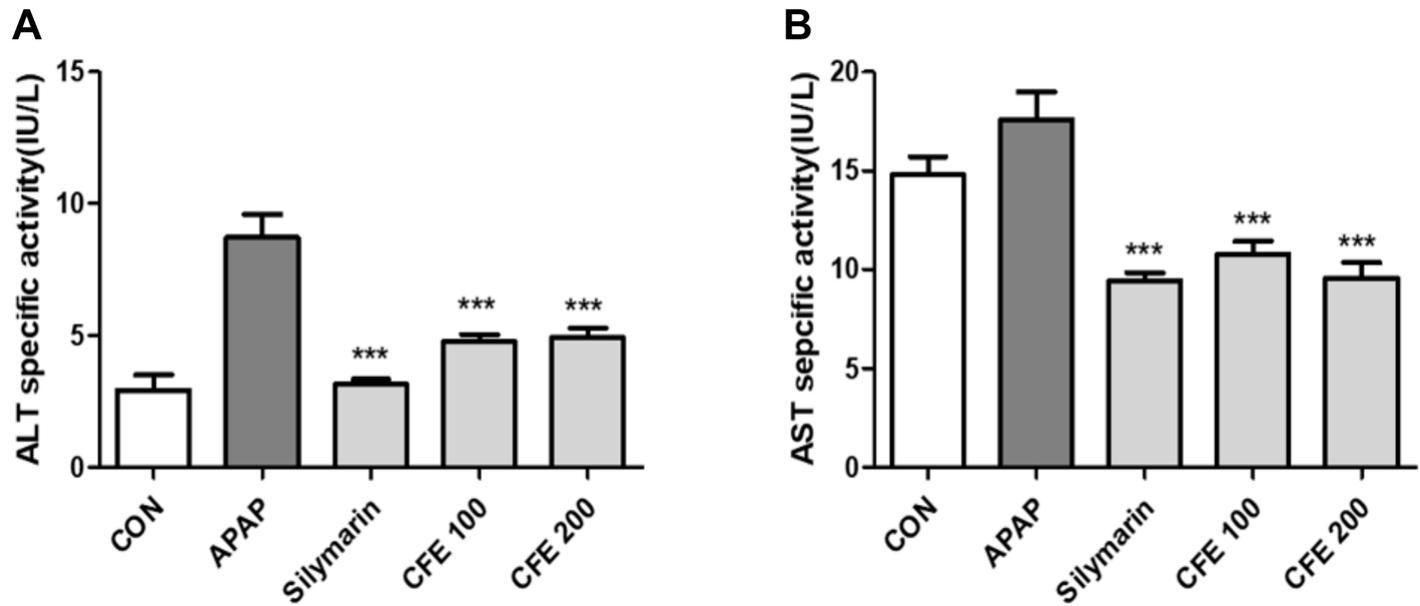
Induction of liver damage by APAP after oral administration of CFE in mice. Compared with the control group, the APAP treated group showed higher ALT (**A**) and AST (**B**) levels. The increased ALT and AST levels were shown to be reduced by CEF treatment. Values are expressed as mean ± SME (*n* = 2). ***p < 0.01 vs APAP. Sily: Silymarin.

The blood ALT and AST concentrations in each group are shown in Fig. 5. In the control and APAP treatment groups, the ALT activities were 2.9 IU/L and 8 IU/L, respectively, confirming that the ALT activity increased because of APAP treatment. These results were similar to the values shown in other studies using the same kit, confirming that APAP induced acute liver damage [24]. The CFE 100 and CFE 200 treatment groups also showed 4.7 and 4.9 IU/L, showing relatively lower ALT concentrations than the APAP treatment group. The blood AST activities were 14 IU/L and 17.5 IU/L in the control and APAP treatment groups, respectively, confirming that the AST concentration increased because of the APAP treatment. In the silymarin treatment group (used as a control group), the AST activity was 9 IU/L; in the 100 and 200 μg/ml CFE treatment groups, the AST activities were 10 and 9 IU/L, respectively, which were relatively lower than that in the APAP treatment group. Thus, according to these findings, CFE reduced blood ALT and AST activities, which were increased by APAP treatment (Fig. 5A and 5B). According to the above results, CFE has a liver protection effect in the mouse model of acute liver damage induced by APAP.

### Effects of CFE on IL-6, an Inflammatory Cytokine, the Secretion of Which Is Induced by APAP in Mice

**Fig. 6:**
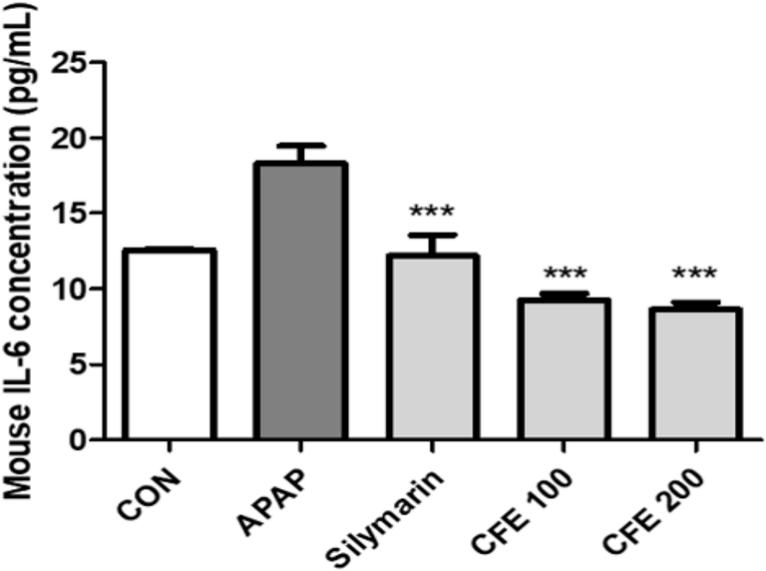
Induction of liver damage by APAP after oral administration of CFE in mice. Levels of the inflammatory cytokine IL-6 were measured after oxidative stress. Values are expressed as mean ± SME (*n* = 2). ***p < 0.01 vs APAP. Sily: Silymarin.

The IL-6 concentration was measured in serum obtained from mice. The control and the APAP treatment group treatment groups showed IL-6 concentrations of 12 pg/ml and 18 pg/ml, respectively, confirming that the IL-6 concentration increased. The Silymarin treatment group had a 12 pg/ml concentration, which confirmed that IL-6 levels decreased. The CFE 100 and 200 treatment groups showed IL-6 levels of 9 and 8 pg/ml, respectively, which were relatively lower than those in the APAP treatment group and relatively lower IL-6 levels than those in the positive control group (silymarin treatment) (Fig. 6). These results indicate that CFE has the effect of suppressing inflammation and protecting the liver by regulating cytokines related to the inflammatory response.

## Conclusion

In this study, our research showed that Loliolide, an important component of CFE, could protect APAP-induced liver damage due to its anti-inflammatory effect. CFE and Loliolide reduced the activity levels of ALT and AST, which are liver damage enzymes induced by APAP, and suppressed TNF-α, IL-6, and MAPK activation to downregulate inflammatory responses. Therefore, CFE can be sufficiently utilized to improve liver function with anti-inflammatory effects derived from marine natural products and hepatoprotective effects. These findings will provide a new perspective for developing functional foods using seaweed to improve liver function. Further studies should be conducted to evaluate the effects of Loliolide in animal models and single and repeated toxicities for its development as a functional food.

## Figures and Tables

**Table 1:** PCR primer sequence list.

Origin	Oligonucleotide sequence (5’→3’)		
	Target	Forward (5’-3’)	Reverse (5’-3’)
Human	GAPDH	ACACCCACTCCTCCACCTTT	TGCTGCAGCCAAATTCGTTG
	TNF-α	AGCCGCATCGCCGTCTCCTA	CAGCGCTGAGTCGGTCACCC
	IL-6	TCGAGCCCACCGGGAACGAAA	AGGCAACTGGACCGAAGGCG

**Table 2:** In vivo group.

Control	APAP	Positive Control	CFE 100	CFE 200
0.9% Saline	0.9% Saline + APAP 300 mg/kg	Silymarin + APAP 300 mg/kg	*CFE* 100 mg/kg + APAP 300 mg/kg	*CFE* 200 mg/kg + APAP 300 mg/kg
